# Foreign

**Published:** 1840-10-31

**Authors:** 


### FOREIGN.

*Mr. Walker on the treatment of Iritis.*—The treatment of iritis should be conducted with energy and judgment. If the case be seen early, and before the adhesive process is properly established, there is good reason to hope that our efforts to arrest the diseased action will be successful. The chief agents to be employed to effect this end are blood letting and mercury. The relative merits of these two powerful agents are by no means clearly established. Some practitioners are of opinion, that one may be safely employed to the exclusion of the other. My own opinion is, that, as a general rule, their employment in conjunction is the safest practice. Still I have no doubt that many cases are curable with either. Thus, I have often known, in cases in which I had prescribed blood-letting to be followed by mercury, that the disease had yielded almost immediately after the abstraction of blood, and before the mercury had had time to produce any perceptible effect on the constitution. In other instances, it has frequently happened that after blood-letting had been practised several times, and the disease yet continued to progress, no sooner were the peculiar effects of the mercury visible than the morbid action at once ceased, and a rapid improvement resulted. I think blood-letting is more likely to be effective, when resorted to previously to the effusion of lymph or pus; and, on the other hand, that when effusion has taken place, mercury will be found to be the most efficient remedy.

The quantity of blood to be withdrawn, and the method of obtaining it, will depend upon the peculiarities of the individual case, more particularly the strength of system of the patient, and the intensity of the local and general symptoms. If the patient be tolerably robust, the vascularity considerable, and there be much febrile excitement present, then the free abstraction of blood from a vein ought to be practised, the quantity to be regulated by the effects produced on the system, and the appearance of the eye, during the operation. Should the inflammatory action not be materially diminished on the following day, and the strength of the patient not materially reduced, venesection may properly be repeated. If this, however, should appear unadvisable from the condition of the patient, we may recommend the application of a number of leeches around the orbital region, or the cupping apparatus to the temple. The number of repetitions which may be requisite will depend so much on circumstances, as to render it difficult to lay down any general rule on this point. In a case of less severe character, or in which the constitution of the patient is delicate, venesection may be superseded by cupping, or the application of leeches.

The employment of mercury in iritis, is second only to blood-letting. Its administration should be commenced immediately, because it is uncertain what length of time it may be necessary to continue it, before the peculiar effects which it produces will be observed. In some persons, we know that these effects are readily produced, while in others it is exactly the reverse. In an acute case, where it is desirable to produce these effects without much loss of time, I usually find that doses of five or six grains of calomel, given every three or four hours, are most to be depended upon. In less urgent cases, I usually prescribe ten grains of blue pill to be taken every four or six hours. In delicate persons, and in children of strumous constitution, and in those individuals who are more readily affected by mercury, I generally substitute small doses of calomel, or of the hydrargyrum cum cretâ, for the more energetic formulæ before mentioned.

On the other hand, if a difficulty be experienced in producing the full effects of mercury, then the employment of mercurial ointment by friction should be resorted to. We are generally advised to rub it on the inside of the thighs or arms, but I think I have found it to be more successful when rubbed underneath the jaws.

The length of time which it may be necessary to continue the exhibition of mercury, must be regulated by its effects both on the system at large and on the condition of the eye in particular. It is obvious that after pytalism, or considerable ulceration of the gums, has been produced, the remedy must either be discontinued, or its exhibition be considerably restrained. If, with these effects on the salivary system, the condition of the eye be simultaneously improved, as denoted by the diminished vascularity, the partial absorption of the effused matter, the gradually-dilating condition of the pupil, and the diminution of pain, then the mercury may be at least temporarily discontinued, its resumption to be regulated by the cessation or continuance of the morbid action which led to its original use.

The next remedy of importance, after blood-letting and mercury, is the local use of belladonna. You cannot begin the employment of this remedy at too early a period of the treatment, because, if adhesions are not already formed, the dilatation of the pupil will be easily accomplished; and if the adhesive process is established, then, so soon as the adhesions begin to yield, the belladonna will produce its peculiar effect on the iris. You will perceive, therefore, that it is most important to have recourse to it in the very earliest stage of iritis, if possible before adhesions are formed; because, otherwise, it will not produce the proper effect until the morbid action is subsiding, and therefore when it is less needed.

The two most material consequences we have to fear, in a case of iritis, as I have before intimated, are a fixed contraction of the pupil, and a deposit of lymph on the capsule. In some cases of iritis, the pupil becomes actually obliterated, by which I mean the fibres forming the pupillary margin of the iris are approximated, or at least agglutinated together by the intervention of coagulable lymph, which is poured out from the surfaces of the iris, during the state of extreme contraction of the pupil. More commonly, however, vision is impaired from the deposition of lymph on the clear, transparent texture immediately behind the pupil. Sometimes this deposit becomes speedily organised, and remains permanently adherent to the capsule; on other occasions, it becomes absorbed, and the transparency of the capsule is restored. It may be that the deposition of lymph results from the extension of the inflammatory action to the capsule, but it is more commonly supposed to be secreted from the surface of the iris itself. If this latter opinion be correct, we perceive the importance of procuring a dilatation of the pupil, so as to remove the iris to a distance from the centre, and towards the circumference of the capsule. The use of belladonna, then, so as to maintain an artifical dilatation of the pupil, is a matter of importance in the treatment of iritis.

And here you may perhaps be inclined to ask for an explanation of the peculiar effect of belladonna on the iris. This involves the question of the mobility of that texture. Now, although there is a difference of opinion as to the cause of the mobility of the iris, yet the view generally entertained is decidedly in favour of muscularity. The motions of the iris, then, I venture to state, are accomplished by two sets of fibres; a set of circular fibres, surrounding the margin of the pupil, and a set of radiated fibres proceeding from the former towards the ciliary or outer margin of the iris. The actions of these opposing muscular fibres are regulated by branches from the third and fifth pairs of nerves, through the ophthalmic ganglion; the former exciting the radiated, the latter the circular fibres. Other branches of the fifth are also freely distributed to the iris, palpebræ, and eyebrows; these latter are the medium of communicating the narcotic effect of the belladonna to the circular fibres of the iris, which, by its influence, becomes paralysed; and thus the radiating fibres, having no antagonist, cause an expansion of the pupil, by drawing the substance of the iris towards the ciliary margin. This appears to me the most rational explanation that can be given of the action of narcotic substances upon the iris. I cannot here give more than this general statement of my views on this subject, and for further information I must refer you to my physiological treatise on the eye.

The ordinary mode of employing belladonna is, by freely rubbing the moistened extract on the outer surface of the palpebræ, and on the skin of the eyebrows; and a portion should also be spread on a piece of soft linen, and laid over the whole orbital region. It should be kept constantly applied, and often renewed, particularly in the evening, because, during sleep, there is the greatest tendency to contraction of the pupil. Some practitioners introduce a few drops of a solution of the extract upon the conjunctival surface; but this is not so proper in the acute stage of the disease, since it increases irritation, and is, probably, not more efficacious than when applied to the cutaneous surface.

Belladonna is the substance most to be depended upon for producing dilatation of the pupil; but there are two or three other vegetable productions which have a similar effect, such as hyoscyamus, stramonium, and laurocerasus. These substances have been long known to produce this peculiar effect on the iris, but the subject was first brought before the profession in a methodical manner by Professor Himly, in 1801.

Blood-letting, mercury, and belladonna, then, are the remedies on which we must chiefly rely for the successful treatment of iritis. Other remedies, however, are sometimes had recourse to. Within the last few years, spirit of turpentine has been successfully employed, particularly in cases in which mercury is considered objectionable. Mr. Hugh Carmichael was the first to suggest this remedy, which he prescibed in drachm doses, given with almond emulsion; and it appears that the disease has been frequently cured, and with as much facility, by it as after the use of mercury. If mercury be sometimes an objectionable remedy, turpentine would seem to be at all times a disagreeable one; and, as mercury appears to be more certain in its effects, it is hardly likely to be superseded by turpentine; the latter medicine seems to operate by producing a considerable amount of irritation in the urinary organs; in fact, its action is that of a counter-irritant. It is doubtful if mercury act in a different manner; its superior value being probably owing to the more powerful and lasting effect produced on the salivary system, and through it on the system at large.

If, during the progress of the disease, the patient should experience much pain in the organ or neighbouring textures, which, when it occurs, is usually of a neuralgic character, the employment of opium, both internally and by friction, in the manner before explained, when speaking of the treatment of sclerotitis, will be advisable. Indeed, opium may be administered in combination with the mercury which is exhibited, except there should be some particular reason to the contrary.

Purgatives, diaphoretics, and other remedies, will, under certain circumstances, be necessary; these I need not dwell upon, as they are sufficiently obvious.

Local applications are of little or no use in the acute stage of iritis, except, indeed, such as are of a sedative character, as the extract of belladonna, mercurial ointment, with opium, and the like.

### *Chronic Iritis.*

The observations already offered are more particularly applicable to the acute form of iritis. If the disease have existed for some considerable time before we see the patient, or if it be of a less active character, the symptoms may be somewhat modified; the vascularity, although possessing the peculiarities before pointed out, may be of trifling extent, the irritation less severe, the patient complaining of little but imperfection of vision. In this state the pupil will be more or less contracted, motionless, and of irregular form; an opaque deposit will probably occupy a considerable part of the capsule immediately behind the pupil; or, if the disease be in a still more advanced stage, we may find that the iris has a puckered and very irregular appearance, some portions of its surface bulging forwards, and others drawn backwards. This last condition probably depends upon extensive adhesions to the capsule of the lens, more or less complete at various points, (synechia posterior,) and is not often witnessed except in conjunction with a morbid condition of the deeper-seated textures, particularly the choroid. In many of these cases, the aqueous humour being no longer secreted in sufficient quantity, the iris is gradually pushed forwards, and with it, probably, the opaque capsule and lens, until it comes in contact with the inner surface of the cornea, when the anterior chamber is obliterated, constituting the condition termed *synechia anterior*. In this event, however, I do not imagine that the iris becomes adherent to the cornea, as some suppose, except there has been actual disease of the latter texture, the adhesive process having terminated long before this stage of the disease arrives.

In other instances, in addition to contracted pupil, and opaque capsule, I have occasionally noticed a decided vascularity of the iris itself, vessels being clearly traceable from the sclerotica on to the iris, forming a plexus over its entire surface, and that of the opaque capsule. In some rare cases, too, I have witnessed effusion of blood into the anterior chamber; this has generally occurred in cases in which the disease has been protracted, and liable to frequent exacerbations.

In chronic iritis, then, the vascularity being inconsiderable, and the morbid process of a slow and inactive character, the treatment need not be of that energetic character as to call for much blood-letting. In some instances, it may be necessary to cup the temple, or to apply a few leeches around the eye. If there be scarcely any vascularity, and we have but the effects of the morbid action to contend with, then blood-letting will not be of any service, and we must trust more particularly to the internal exhibition of mercury, the local application of belladonna, and the use of counter-irritants.

Mercury should be given in smaller quantities, and not so frequently repeated as in the acute form of iritis, as the object of the practitioner should be not to create a violent, but a more continuous effect on the system. With this view, from five to ten grains of the mercurial pill may be given night and morning to an adult; and to children, one or two grains of calomel, or the hydrargyrum cum cretâ, at the like intervals. This treatment it is usually necessary to continue for some time before any perceptible change in the condition of the eye is produced, or before the system evinces any decided effect from its employment.

The extract of belladonna should be applied every night to the eyebrows and lids, and a small portion should also be introduced within the margin of the lower lid, so as to come in contact with the conjunctival surface; or a strong solution of the extract may be applied to the conjunctiva, by means of a camel-hair brush, once or twice a day. I am not sure that belladonna has any very powerful influence in separating adhesions when once formed; but its application may be useful in causing a dilatation of the non-adherent portions of the pupillary margin, and may thus be serviceable in preventing new adhesions forming. Indeed, its employment may tend to throw considerable light on cases which appear obscure and uncertain; since, if the pupil dilate irregularly, it is a clear proof that there are partial adhesions with the capsule, and that inflammation and effusion have existed; and, if the adherent surfaces are separated under its use, it will be likewise a proof of the subsidence of the morbid action, and will assist in determining the propriety of continuing, or otherwise, the remedies best adapted for its subduction.

Counter-irritation I am inclined to recommend in the advanced stage of iritis; it may be produced by the occasional application of blisters, or tartar emetic ointment, to the temple; or a permanent drain may be established by means of a seton or issue, according to circumstances.

In the more protracted cases of iritis, particularly such as are accompanied by an enlarged and varicose condition of the vessels, I have found very beneficial results from the application of stimulants to the conjunctival surface of the lids. These can only properly be resorted to after the acute stage has passed away. I have ascertained, by careful experiments, that their use in acute internal ophthalmia is not to be commended. In one case of iritis, which, although it had existed for several weeks, was attended with considerable vascular excitement, I applied the nitrate of silver in the usual manner, and found that its use was followed by an effusion of puriform fluid into the anterior chamber; the effused fluid, however, gradually became absorbed, and, although its employment in this case was not prejudicial, since the eye ultimately recovered completely, yet I could not observe any indication for its future use. In a more advanced stage, however, I am quite satisfied that it will often prove a valuable agent.

*Syphilitic Iritis.*

I have already stated that syphilitic disease often extends to the iris, and give rise to acute inflammation of its texture. This is a very frequent occurrence, so much so that when we find iritis among a certain class of individuals, we immediately inquire if there are any other symptoms of syphilis, either primary or secondary. Iritis is most commonly met with among the secondary or constitutional symptoms of lues, such as ulceration of the throat and eruptive disease of the skin. It is as well that you should be conversant with this fact, so as to be aware of the frequent combination of syphilis with iritis; but I do not know that it will lead to any practical results, since the treatment of iritis, however excited, must always be conducted on the principles I have laid down. Nor are there any diagnostic symptoms which could enable a practitioner to say, by merely examining an eye thus affected, that such a case is syphilitic or otherwise. It was formerly thought that the existence of tubercles and the displacement of the pupil upwards, were decisive of the syphilitic origin of the disease. Such a notion, however, is now completely exploded.

You will find in several of our modern works very elaborate articles on the subject of syphilitic, rheumatic, arthritic, and strumous iritis. The conditions of the system, indicated by these different epithets, render the eye, perhaps, somewhat more disposed to be affected by disease than in persons of a healthy constitution; but that such conditions materially or perceptibly modify the characters of ophthalmia generally, or of iritis in particular, is, to my mind, certainly not established. Moreover, the treatment admits of no further modification, so far as the eye is concerned, than that I have already pointed out, and which relates more to the intensity and duration of the disease than to the exciting cause.

In whatever kind of constitution iritis occurs, it will require blood-letting and mercury, if the activity be considerable; but, in a person of delicate or strumous constitution, these remedies should be sparingly employed, and their use speedily followed by the exhibition of quinine and other tonics. If iritis occur in a person of a rheumatic or gouty diathesis, then colchicum and other suitable remedies may be employed in conjunction with those I have before advised.

When iritis is observed in very young children, it is, probably, usually in connection either with a strumous or a syphilitic taint; and, in these cases, I have generally found, if the pupil have not become fixed, and the deposit on the capsule organised, before the patient has been brought, that the symptoms have yielded to the exhibition of small doses of calomel, or hydrargyrum cum cretâ, two or three times a day, and the local application of belladonna. Leeches will also be proper, if the vascularity be considerable. Iritis is not very commonly met with in infants, but this occasionally happens. During the last two years, I have witnessed four cases between the ages of four and eight months. In one of these, one eye had been attacked with purulent ophthalmia at the period of birth, from which it had completely recovered under proper treatment; the other eye was attacked with iritis at the age of five months, from which it also recovered, leaving the pupil contracted and irregular, but without any opacity of the capsule. In this instance, it was admitted that the parents had been affected with some form of venereal complaint.—*Lancet.*

---

*Remarkable Arrest of Development in the Organs of Generation, and in the Urinary Apparatus.*—John Battle, aged 24, six feet in height, was admitted lately into the hospital, under the care of Dr. Chowne. He is a native of Clopton, in Suffolk. He was admitted under the name of "Elizabeth," having from his childhood worn a woman's dress, and being considered by his neighbours to be of that sex. On first looking at him, and before he spoke, his features had the general characteristics of the softer sex. His hair was dressed like that of a female; he had extremely little beard or whiskers, the former not being sufficiently strong to leave any appearance after shaving, although the hair is dark. The hair is most scanty on the upper lip and chin, and less so below and behind the angles of the lower jaw. The pelvis at the highest and broadest part was thirty-seven inches in circumference, and was flat in proportion to its breadth. The right thigh and leg were rather smaller, weaker, and shorter than the left. The disparity in the length of the inferior extremities, together with his bending a little forward when he walked, to protect the lower part of the abdomen from the contact of his dress, gave him a peculiar and limping gait. The mammæ were flat and masculine; the papillæ small, with a few hairs around them. Surface of the thorax and abdomen free from hairs; voice decidedly masculine; general health good. He was not aware that there had been any malformation in his family. On the lower part of the abdomen, a little above the pubes, there was absence of the anterior wall of the belly, and of the anterior portion of the bladder, the posterior portion of which fell forwards into the opening, and was united by cicatrices to its margin everywhere, except at its lowest part. The surface of the protruded portion of the bladder had a circumference of about two inches; it presented the mucous membrane, highly vascular, of a bright red colour, and covered with the natural mucous secretion. At some parts the mucous membrane was absent over a small space; this, according to the account of the patient, occurred at different times at different parts, the places being afterwards again supplied with the red mucous covering; the whole of the exposed surface very tender to the touch. At the lower part of the surface of the bladder the ureters opened, their situations were obvious, although it was difficult to distinguish their exact orifices; on the left side there was a protuberance or fold of the bladder just over this part, so as to make the opening still more obscure. The openings were nearly horizontal to each other.

The urine constantly passed in small quantities from the ureters, but at times a quantity, equal to about a drachm from each ureter, would appear to have been retained, and forced out when any bodily exertion was made. The penis occupied its natural situation, and was capable of vascular turgescence, as when fully formed, although in a minor degree. Including the glans it was about two inches in length, and never, at any time, exceeded three. There was no prepuce, except a small fold at the under part of the glans, where it existed in connection with a distinct frænum; the glans was quite exposed. There was no canal constituting an urethra, but a longitudinal depression on the upper side, giving the appearance of a groove; it was covered by a thin pellucid membrane, and continued to the extremity of the glans. The organ at its termination, therefore, seemed bifid, and when the lateral portions were brought together, and made to meet above, the orifice at the extremity corresponded with that of the meatus. The upper surface of the part rested against the projecting portion of the bladder. Between the most posterior part of the organ and the lowest point of the projecting portion of bladder there was no union by cicatrix, as at the other parts, but the appearance of an undefined, rather deep opening. There was no prostate gland, urethra, or corpus spungiosum. As the urethra is naturally a continuation of the lining membrane of the bladder, and is the part on which the corpus spungiosum is formed, the abrupt termination or arrest of development of the bladder at the point corresponding with the pubes, explained the absence of those parts which essentially belonged to the urinary system, although subservient to another; while the corpora cavernosa and glans not essentially urinary, nor derived from the bladder, were present. The scrotum was naturally formed; the testicles were of dissimilar size, the right of the natural size, the left about half the natural size; both, together with the spermatic cords, were more than usually tender on pressure; on either side was a large inguinal hernia; the scrotum and inguinal regions were supplied with hair; it became scanty, however, on each side of the projecting bladder, and finally disappeared above the bladder, and in the line of the linea alba there was none. The anal extremity of the rectum was close to the scrotum, and where the perinæum in well-formed subjects commenced, leaving, therefore, in this case, no perinæum.

On either side of that which, in well-formed subjects, would be the pubis, a bony projection might be felt by the finger, at which point the bony structure of the anterior part of the pelvis terminated; the interspace was supplied by strong ligament; this was perceptible both by external and internal examination. There was no vestige of an umbilicus. At one point of union, between the parietes of the abdomen and the protruding portion of the bladder, there was a cicatrix, but it did not at all resemble the navel, and there were other similar cicatrices all around at the points of union. This patient had lived to the present time subject to all the distressing inconveniences of occasional and painful contact of his dress with the protruded and delicate surface of the bladder, and to the misery of being in a constantly wet and sometimes an excoriated state.

He was seen in his native village by Mr. Muriel, surgeon, of Wickham Market, who, considering that the case admitted of a diminution of some of the patient's distresses by the help of mechanism, for which London afforded greater facilities than the country, procured his admission into this hospital, and he was sent to town for the purpose. The instrument with which he is now provided, possesses the advantage of a shield, protecting him from the painful contact of his dress, and serves as a reservoir to receive the fluid, and to retain it until it may be convenient to him to allow it to be discharged.

In some clinical remarks on this case, Dr. Chowne observed, that although malformations of the class to which this belonged differed frequently in minute points, yet that a general similitude prevailed, not only as regarded the bladder and the parietes of the abdomen immediately concerned, but as regarded, also, other parts involved, whenever male cases were compared with each other, on the one hand, or female cases on the other, but that such malformations occurred much more frequently in the male than in the female.

Although the patient, at present under consideration, had no malformation which could lead to the possibility of doubt as to his being a male, and although his own feelings were fully confirmatory of that fact, yet it could not be denied that his personal appearance, in some respects, had much of the female character, independently of the auxiliary influence of his mode of dress. This showed itself, however, chiefly in the face, the expression of which, generally, was not that of a man; the upper lip and chin were peculiarly deficient of male characteristics, in form, expression, and beard. He was also moved to tears upon slighter occasions than was usually seen to have that effect even in men suffering from illness and present misfortune. The expression of his eye, however, sometimes in the course of conversation, formed a strong contrast with his other features, and his voice was strictly masculine. It was proper to take into consideration, moreover, that he had always associated with women; that with their apparel he had adopted their society and habits from his childhood upwards, and this might have had considerable influence on the physiognomy, not only when at rest, but when in conversation. He was quick of comprehension, and intelligent for his sphere of life and secluded habits.

The peculiarity of his gait, although principally owing to the shortness of the inferior extremity of one side, and to the stooping position to prevent friction from his dress, was, without doubt, augmented by the absence of bony union at the symphisis pubis. First, the pelvis was necessarily less firm as a support for the upper structure. Secondly, it was thrown into what was an irksome, and would soon be a painful position, when resting with its superincumbent weight upon either femur. Thirdly, the absence of bony union occasioned greater breadth of pelvis, throwing the acetabula, and consequently the femora, further apart; all which circumstances conspired to make walking both difficult and peculiar, increasing in a greater degree the lateral movement, and decreasing the firmness. The colour of the protruded surface of the bladder was pretty similar at all times, namely, a bright red; this, however, varied in different cases, and had in others been described as red, either pale or lively, or dark and almost liver-coloured. In this instance, it resembled in colour a portion of prolapsed rectum as frequently seen in children. The surface was rather uneven, in this also corresponding with what is most common; it was frequently described as wrinkled, granulated, or tuberculated, and as having the appearance of fungous or excoriated flesh. The texture had been described as spongy, membranous, vascular, pulpy, or parenchymatous, with glandular pores.

The extent to which the surface protruded, varied very considerably with the position, and according as there was straining from any cause. When the patient had been sometime up, it had three times the volume which it presented when he had been some time reclining on his back.

Position made the same difference in the degree to which the herniæ protruded: when Battle was examined in the morning in bed, the herniæ were very much reduced, the inguinal regions and the scrotum appeared almost natural, and their contents might be accurately examined; but after he had been some time up the herniæ descended, the inguinal regions became full and very much distended, the scrotum drawn up and altered in its appearance, and the parts within scarcely, if at all, distinguishable. It was necessary in all cases to keep these facts in view, or the judgment and diagnosis might be very much embarassed. It was stated by some writers on the subject, that in such patients the region of the pubis was particularly liable to accumulations of fat, especially in the lines of the spermatic cord giving the appearance of hernia. In almost all cases of malformation similar to Battle's, the description of the virile member corresponded with the appearances observed in his case; besides the outward general characters, the part had the appearance of being retracted; and this, to a certain extent, was the case, and was owing to the greater width of pelvis permitted by the ligamentous union of the ossa innominata at the pubis: the greater separation of these bones at the anterior part caused the origins of the corpora cavernosa to be at a greater distance from each other; in order to unite, therefore, they directed their course more inward and less forward than common, and a larger portion of the structure was, consequently, retained within. The absence of bony structure and the substitution of ligamentous union was very general, if not constant, in this kind of malformation; it was the case in the young female patient, some time since in this hospital, admitted for a deformity of the same class. It was particularly necessary that this fact should be kept in mind, especially when the sufferer was in a sphere of life which might subject him, or her, to engage in manual occupation. It was obvious that such occupation should be of the lightest possible description, as other labour could not be performed without great increase of suffering, inevitable injury to the person, and most probably diminution of the period of existence.

We found in Battle's case that which might be regarded as the defect of formation most common in similar cases. At that point, immediately below the inferior margin of the exposed bladder, as already stated, there was not any cicatrix, but, instead, the appearance of a deep penetrating wound; within this, but very near the surface, the vasa deferentia terminated abruptly. This was found to be common in examples which had afforded opportunities of post mortem examination. In the case under consideration, the characteristic secretion which at times passed from it, indicated the proximity and termination of these vessels. The anal extremity of the rectum in malformations of this character was very commonly, as in Battle's case, thrown unusually forward; but fortunately, he did not, as was very common, suffer any inconvenience of constipation, or otherwise, from it; and there was every reason to infer that the intestinal canal did not, as occasionally happened, participate in the defect of formation. In different instances the rectum had been found to be extremely narrow on the one hand, or extremely dilated on the other; or, together with other parts of the canal, altogether wanting.

A case was on record, in which it was stated that there was no colon, cæcum, or rectum; that the ileum terminated in a fleshy pouch, and that from this a narrow tube supplied the place of the rectum; it might, perhaps, however, be suspected, that the fleshy pouch on the one hand, and the narrow tube on the other, were, in fact, rudiments of the cæcum and rectum, however imperfectly formed. The umbilicus, in this kind of malformation, was subject to greater variety than the other parts generally involved; yet its position observed a general rule, and was most commonly at the upper edge of the protruded bladder, the cicatrix being more or less blended with that of the bladder and abdomen. In some instances it occupied its natural position. In the case of Battle there was nothing that could be recognised as an umbilical cicatrix, that which was nearest to the umbilicus was flat, smooth, and glossy.

In examples where a similar state had existed, there had arisen a doubt whether there had ever been a vascular connection between the fœtus and the mother; but such suspicion could not now be encouraged.

In whatever degree there might be a deficiency of masculine character, it might, perhaps, be fairly accounted for, by taking into consideration the condition of those parts on which the physiological development of male peculiarities depended, with which might be included the spermatic cords. On the right side, the testicle, although of the normal size, was unusually tender to the touch; on the left side, it was not more than half the usual size, and was, also, morbidly sensitive. On both sides the spermatic vessels were tender under pressure, and obviously unfavourably placed; the adjacent parts being in a state of malformation and morbid excitement, partly from the immediate vicinity of the protruded bladder, partly from the irritation of effused urine, partly from the presence of large herniæ; and added to these, it was more than propable that some alteration in the anatomy of the abdominal ring, consequent upon the unusual structure of the pelvis, might conspire to embarrass the names of the spermatic cords in their functions, and the spermatic arteries and veins in them.

It appeared to be extremely probable that such a state of things existing from childhood, and being of a permanent character, might impair the healthy tone of the parts, and so weaken their physiological influence on the system.

Battle appeared never to have engaged in any other occupation than making a few straw hats for men, and he was from his corporal infirmities incapable of any but the slightest kind of employment, requiring little strength, little motion, little strain upon the body, and not long confinement in any one position.—*Ibid.*

---

*Case of dangerous Uterine Hæmorrhage in which Transfusion was successfully employed, with some observations on the more frequent expediency of that operation.* By Richard Oliver, M. D.—On the 26th of June, 1837, I was called to attend the wife of John Cook, a weaver, living at Eden Place, about a mile from my house. She had been attended by a midwife, and had given birth to a child at its full time in the course of the previous night. The patient was about forty-two years of age, and this was her seventh child. I found her at 6 A. M. in an exceedingly exhausted condition. Blanched by a profuse hæmorrhage, which no adequate means had been employed to suppress, but which had now ceased, she was lying on her back in a state of imperfect consciousness, with the pulse at the wrist barely perceptible, now and then moaning lowly, and casting about her arms. About half a glass of rum, with a little water, was immediately given to her, and this, with a few spoonsful of beef-tea, was repeated two or three times at intervals of about twenty or twenty-five minutes. After each dose she appeared to be a little refreshed, but upon the whole the symptoms of collapse were gaining ground. About half past 7 o'clock brandy was substituted for the rum, and the dose was increased to an ounce and a half, with the addition of a drachm of aromatic spirit of ammonia, and a few drops of tincture of opium, to every second or third portion. The same deceitful promises of reaction were succeeded by the same progressive indications of sinking, until at length, about 1 P. M., she became quite unable to swallow. The pulse at the wrist and in the carotids had not been perceptible for more than two hours and a half, and the coma was now complete. Under these very unpropitious circumstances I determined upon transfusion, with little hope of success, and with no small compunction for having thus afforded the operation so little fair play. At half past 1 P. M. I was provided with the apparatus necessary for performing it; and having obtained a willing supply of blood from three of the patient's kind-hearted neighbours, I opened a vein at the bend of her arm, and, with the assistance of two of my professional friends, Mr. Bowman, surgeon of this place, and Dr. Henry Lonsdale, now Demonstrator of Anatomy in Queen's College, Edinburgh, I proceeded cautiously and steadily to introduce it.

I had first taken care to see that the instrument was in proper order, and particularly that I should have the syringe and its tubes free from air. After one or two gentle strokes with the piston, made with a view to ascertain this point, I found that the cup attached to the apparatus was so small that it could not be safely used. Unless the piston was elevated very slowly, and the blood was supplied very steadily to the cup, there was great risk of introducing air into the cylinder. But finding that, by taking a common basin to receive the blood, and by drawing it up thence through the bottom of the syringe, I could obviate this danger, I laid the cup aside altogether. With this simpler arrangement I passed syringeful after syringeful into her exhausted veins, pausing from time to time to mark the effects, and anxiously watching for some glimmering promise of the return of energy to her heart. On a moderate computation we had already transfused twelve ounces of blood, and she still lay pulseless and perfectly insensible. The respiration, however, although faint and low, was distinct and regular; so that, however small the amount of blood in her system might be, there was still some undergoing aeration in the lungs; and in gradually augmenting its quantity, we might possibly contribute to raise her vital powers, by enabling a larger portion of it to reach the nervous centres. We could not discern the heart's pulsations, but we might be quite certain that it did beat, and that the general circulation, although thus imperceptible, was still actually carried on. We had yet obtained no assurance of improvement, but it was pretty evident that, by proceeding cautiously, we neither had done, nor could do any harm. Without this expedient the poor woman's death was inevitable, and but too probable, we then thought, even with it; so, disregarding the cautions given upon this point, we determined to go on. Steadily and slowly the blood was introduced as before, until at length we imagined that the pulse became faintly perceptible in the arm; and slight as it was, this intimation of the heart's increased power gave us no small encouragement. After persevering for a few minutes longer, we had the very perfect gratification of witnessing not only the complete restoration of the circulating power, but the return of consciousness, and of the ability to speak.

It is unnecessary to advert to the subsequent details of the treatment and of the recovery, farther than to mention that she went on very favourably, and in a few weeks was moving about in her family as usual. She remained for some time longer rather weak and delicate, but beyond an occasional slight headach, and a tendency to constipation and flatulence, she suffered from none of the more prominent and distressing symptoms which ordinarily ensue after serious losses of blood, and she has long been, and still remains, in very good health.

With respect to the quantity of blood introduced in this case, I am not able to speak with absolute accuracy; but I feel quite certain that I am below the mark in mentioning twenty-two ounces. From each of the three individuals who supplied the blood, we took at the least an average of twelve ounces; and although we did not attempt to measure the amount of it, I am perfectly satisfied that, at all events, not more than one-third of the whole was lost by coagulation, and by being thrown upon the ground in adjusting and preparing the instrument.

In looking over a few of the observations that have been made upon the subject of transfusion, I have been struck with the reasons which are assigned for the directions given as to its performance. Apprehensions are expressed lest the power of the heart should be overwhelmed by too sudden or too copious a supply of the borrowed fluid,* and lest the exhausted system should be unable to appropriate it; and as the first circumstance is not only conceivable, but quite possible,† the apprehension may be to a certain extent a just one. But there can be no reason for allowing this consideration to deter us from making a fair use of the remedy; and I am very much inclined to think that want of success may have resulted from too highly estimating this supposed hazard; for although cases of its failure may not have been unfrequent, I have not yet been able to meet with any proofs of its ever having been positively injurious. The merits of the operation, I believe, have not yet been fairly tested. The whole of the symptoms immediately consequent upon excessive hæmorrhage, whether those which present themselves in cases of leipothymia from any other cause be so or not, are referrible, I presume, for the most part, to a diminution in the supply of arterial blood to the cerebro-spinal axis; and, certainly, when the vascular system has thus suffered an enormous draining, it is almost needless to say that there can be no more direct and obvious mode of counteracting the consequent mischief, and of restoring or upholding the supply of nervous influence which is requisite for the sustentation of life, than to refurnish that system with blood. So long as the respiration continues regular, however feeble, and, which I take to be only another form of expression for the same thing, so long as the right cavities of the heart are able to forward blood to the lungs in a corresponding ratio, that blood will be made fit for its office, and every drop added to the arterial current will augment by so much the probabilities of eventual success, provided the symptoms are merely those of inanition, and that no incurable lesion has pre-existed, or has been produced by delay.

Although the persistence for a short time of the heart's power of action, particularly that of the right auricle, after the irritability of the rest of the system has ceased, is such as to have obtained for that viscus the designation of *ultimum moriens*; it is not less certain that the exercise of that power is essentially dependent upon the presence of its natural and appropriate stimulus; and the influence of a vast variety of circumstances in modifying its action completely shuts out the opinion advocated by Haller,‡ that its motion is solely the

* "But if too large a quantity be injected, it may so oppress the right side of the heart, as to impede the circulation."—Mr. J. H. Green in Lancet of December 12, 1829. Vol. i. p. 372.

† This had been noticed by former observers; but is well attested by Dr. John Reid in his article on the Heart in the Cyclopædia of Anatomy and Physiology, vol. ii. p. 608.

‡ "Is motus est insitus, nec a cerebro advenit, nec ab anima, cum in mortuo animali, cum in corde result of a property exclusively appertaining to itself, and not in any way dependent upon the condition of the nervous system. In any case, therefore, where the blood is dangerously deficient in quantity, transfusion would appear to be available, not merely in the way of supplying the heart with that direct stimulus which is indispensable for its immediate action, but also by maintaining its contractility through the sustained energy of the nervous centres; and the case which I have just related may serve at least to show from how low a pitch the powers of the system may be effectually raised, when ordinary means have proved unavailing, whilst even thus finely spun, the thread is yet unbroken which connects the inter-dependent functions of the heart, lungs, and brain.

The scepticism which insinuates that recoveries might have taken place in many instances whether the operation had been resorted to or not, expresses but a negative objection to its use; and we have yet to learn that the cautious introduction of blood can lessen the chances of recovery when the balance is thus dubiously wavering between life and death. In those cases where a very small amount of blood has been found sufficient to turn the scale, and where it is not improbable that the patients might have rallied without such assistance, the only philosophical objection that has yet been raised against the practice, viz. the risk of oppressing the enfeebled heart, must well nigh have reached its vanishing point; whilst instances such as that which I have detailed sufficiently attest its value under circumstances of a widely different character.

Believing that a very erroneous estimate has been put upon this operation, and that its alleged risks have been greatly exaggerated, I am anxious to see it placed upon a footing where the advantages which it really presents may be fairly dealt by, and to hear of its being more frequently resorted to in those fearful cases which but too commonly occur, particularly in obstetrical practice, to peril, it may be, some life of more than ordinary moment, and to harass the mind of the medical attendant with an anxiety almost insupportable. I cannot help thinking that if the measure were adopted with less hesitation, and much earlier than has been usual, there would be less of this. But whilst practioners, on referring to authoritative systematic works for information upon this subject, meet with little more than doubts as to its expediency, or something like a specious pretext for mistrusting it, it is not likely that we shall speedily hear of its coming into any thing like general use; unless, indeed, Dr. Blundell should resume its advocacy, or Dr. Ferguson, or some other practitioner possessing ample opportunities for observation, and proceeding with a reasonable boldness, should determine on data sufficiently complete, the exact amount of credit due to it as a therapeutical agent.

Dr. Davis, in his Obstetrical Medicine, states, that "experience appears to sanction the presumption that the capacity of the arterial system, considered in connection with its influences on the nervous and respiratory systems, is much too limited to make it probable that it should ever, by any variations or modifications of future trials, be brought to admit and to adopt for its own purposes, within the period of an hour, or even of some fractional proportion of that time, sufficient quantities of foreign blood to insure the recovery of one woman in a hundred, and so serve eventually to promote the re-establishment of the practice of transfusion."—P. 1066.

Now when we find in a work of this imposing character, where we might expect to meet with something like sound reasoning on a subject of this kind, that a vague presumption is thus put in the place of a legitimate philosophical inference, it cannot be denied that the merits of the remedy have been prejudged.

Mr. Lizars, on the other hand, in his System of Practical Surgery, says, after describing the mode of performing the operation, and mentioning one or two drachms as the quantity to be introduced, "the blood thus propelled into the exhausted circulating system re-excites the heart, and puts the springs of life into action. It is only necessary to inject a few drops of blood to stimulate the heart; and probably the saline solution, which was so extensively and so beneficially used in cholera, is preferable to human blood."—P. 126.

This is, no doubt, a more encouraging view of the matter; but if it were found on proof that "a few drops of blood" did not generally "stimulate the heart," and that even "a drachm or two" very frequently failed "to put the springs of life into action," the reputation of the remedy would be more apt to suffer eventually from the false expectations which such an inconsiderate assertion is calculated to excite, than from any direct hostility to its employment.

If I should ever again have occasion to resort to this operation, I would use a much simpler instrument than that which is termed the transfusing apparatus, and one which might very conveniently be in the hands of every practitioner. A small curved silver tube for insertion into the vein, having a transverse flat collar to keep it steady, is of course indispensable. To this a light, straight, brass stop-cock should be adapted; and, when required, a soft bladder tied upon the end of the stop-cock would serve for the reception of the blood. Mr. Syme, I believe, recommends something like this in his Elements of Surde pectore avulso supersit, neque a voluntate aut incitari passit aut tardari."—Haller, Primæ Lineæ Physiologiæ, sect. cii.

gery. In this way the blood could readily be pressed downwards into the vein, without any risk of introducing air. The advantage of a pocket instrument of this kind over the more cumbrous and elaborate machinery which very few people are in possession of, and which is not unlikely to be at an inconvenient distance when most urgently wanted, is sufficiently obvious; more particularly as its constant readiness would very probably lead to its more frequent use.—*Edinburgh Medical and Surgical Journal.*

---

*New Instrument for Tapping.*—Dr. Lendrick remarked, a few days since, to the pupils of Mercer's Hospital, Dublin, that he had often, both to the classes of this and Sir Patrick Dun's Hospital, animadverted on the common trocar, which is liable to many objections, especially if used in cases of ascites. It is altogether a rude and clumsy instrument, and can seldom be rendered sharp, except by a very skilful cutler: the canula also rarely fits it. For these reasons it enters the parts with difficulty, and such force becomes necessary, that the viscera are sometimes reached and injured. The canula is liable to be blocked up by omentum, or, if sharp, (and otherwise the canula will not fit,) to wound the vessels of that membrane. For the rectification of these defects many contrivances have been attempted.

Dr. Lendrick showed the pupils a new instrument, made under his direction by Mr. Milliken, of Grafton street. He stated that he was not aware of a similar plan having been resorted to. The instrument is the "exploring needle," on a large scale, as to breadth, being about three inches long, two lines and a half broad, and one line and a quarter deep. It has an oblique handle, like that of a gorget. There is a groove on the surface, and the extremity is lancet-shaped, with only two edges, extending not quite to the shoulders of the blade. The integuments may or may not be divided with a lancet, according to the wish of the operator. The instrument is introduced with great ease, and the penetration of the sac is at once known by the gush of fluid along the groove: the operator, who holds in his hand a catheter, (No. 4 or 5,) nearly straight, and either of silver or elastic gum, immediately passes it along the groove, and its penetration of the sac is simultaneous with the withdrawal of the sharp instrument. The fluid is now allowed to flow through the catheter, pressure with a swathe being applied as the abdomen becomes flaccid. The whole operation may be performed without varying the position of the patient from the recumbent posture in bed. The wound is somewhat valvular. The operator ought to hold the trocar in one hand, and the catheter in the other. The introduction of the second is to be accompanied by the withdrawal of the first. Thus the aperture of the sac is at once hit off.

The operation of paracentesis abdominis was performed this day, (27th July,) at Mercer's Hospital, with Dr. Lendrick's trocar, by Mr. Auchinleck. It was done without any previous incision, the instrument entering at once with the greatest ease, and almost without being felt by the patient. A gum catheter (No. 5) was used; but No. 7 might easily have been introduced. The fluid passed away slowly, and the patient, who was much debilitated, preserved the recumbent posture throughout. One precaution is necessary to be attended to, if a gum catheter be used. After the stylet is withdrawn, the tube is liable to be compressed by the edge of the swathe, and the exit of the fluid impeded. The difficulty is overcome simply by removing the edge of the swathe from the orifice. It is preferable that the catheter should be furnished with two apertures.—*Lon. Med. Gaz.*

---

*Suspension of Life.*—"But it is not during their embryo state merely, that the vital actions of living beings may be suspended by the deficiency of external stimuli, and yet their vitality be preserved. Both the vegetable and animal kingdoms afford numerous examples of such an occurrence at all periods of existence, especially among their lower tribes. Mosses, for instance, often appear completely desiccated in dry weather, and seem as if dead; whilst, on the application of moisture, they revive in all their pristine beauty. The curious Lycopodium of Peru exhibits this torpor in a still more remarkable manner. When desiccated by drought, it folds in its leaves and contracts its roots so as to form a ball, which, apparently quite devoid of animation, is driven hither and thither by the wind; as soon, however, as it reaches a moist situation, it sends down its roots into the soil, and unfolds to the atmosphere its leaves, which, from a dingy brown, speedily change to the bright green of active vegetation. The Rose of Jericho is the subject of similar transformations. Instances exactly parallel are furnished by the animal kingdom. The common Wheel-Animalcule is one of the most remarkable, being capable of desiccation so complete as to splinter if touched with the point of a needle, and still preserving its vitality so as to revive when moistened. In animals reduced to a state of torpidity by cold, some vital action usually continues; and such cases cannot, therefore, be adduced under the present head. But instances are by no means rare in which the whole body has been frozen, and vital action has of course been completely suspended, yet without the destruction of the power of renewing them under more favourable circumstances. Lister first noticed that he had found caterpillars so frozen, that when dropped into a glass they chinked like stones, but nevertheless revived; and this statement has been confirmed by Bonnet and others. The Papilio Brassicæ has been produced from a larva which had been exposed to a frost of 0° Fahr., and which had become a lump of ice. Fishes are occasionally found imbedded in the ice of Arctic seas; and some of these revive when thawed. This tenacity of life appears greater, however, in the species which are confined to shallow lakes or ponds, and which have not the power, therefore, of escaping from the effects of cold."—*Mr. Carpenter in the "Cyclopædia of Anatomy and Physiology."*

*Lon. Lan.*

---

*Fatty Liver.*—Fatty liver is most frequently observed in persons who have died from scrofulous tubercles in the lungs; in those, says Andral, in whom the blood has not been efficiently arterialised, and in whom the pulmonary exhalation is greatly diminished. Can it be, he inquires, from the absence of the due separation of hydrogen from the lungs that this compound of hydrogen, fat, becomes deposited in the parenchyma of the liver? This question is well deserving the attention of pathologists, and its solution might lead to important information. The disease has also been observed in some cancerous disorders and in dartrous diseases of the skin.—*Cyclopædia of Anatomy and Physiology. Art. Liver.*

*Ibid.*

---

*On Wildbad in the Kingdom of Wurtemberg.* By Dr. Fallati.—The most comprehensive category under which to include patients who are sure to be relieved, if not cured, at Wildbad, is that of persons who are suffering from gouty dycerasia, or a rheumatic diathesis. I have seen all diseases grow better at Wildbad, which belonged to this class, and were likely to be benefited by warm baths, whatever might be the system or organ in which they made their appearance; and among them were cases in which this could hardly have been expected. This character of disease was the cause of the success of the treatment in a great number of cases of paralysis, and anchylosis, as well as of some cases of hæmorrhoidal, calculous, and other urinary disorders.

It is remarkable that, in particular, local diseases, which either consist of a swelling caused by some morbid deposit, or in which such a deposit, though not visible, may be supposed, decrease rapidly and surprisingly, even when they do not depend on a gouty or rheumatic diathesis.

The baths of Wildbad appear to increase the absorption of morbid deposits more than I could have believed, had I not seen it in my own experience. As exudations of this kind occur in arthritic or rheumatic subjects from internal causes, so they occur in persons otherwise healthy from external injuries, particularly about the joints; and it is for the cure of these surgical cases that Wildbad has always been celebrated. It is the same with the different diseases of the bones depending on such causes, together with abscess, fistula, and stiffness in the neigbouring soft parts remaining after fracture. When the patients are young, and can bear the bath, such cases often form the most brilliant cures.

Those cases of paralysis and paresis which have arisen from any pressure on the corresponding part of the nervous system, whether by extravasation or exudation, are proportionably more easy of cure than others. Yet I have seen marked improvement even in those which arose from a dynamic cause, namely, fright. I particularly recollect the cure of a boy, who, in consequence of spasms in one leg resembling chorea, had lost all strength in it. He had partly recovered its use under the care of Dr. Chelius at Heidleberg, who completed his cure by prescribing Wildbad.

I have found its effects on the organs of reproduction and their functions, particularly in women, such as might have been expected from the properties of the water. In men also I have found that too frequent pollutions, arising from an excessive irritability of the genitals, gave way and become normal; while in a case of incipient paralysis, of the lower extremities particularly, emission, which had been absent for months, returned after the use of the baths.

In conclusion, I have to make a remark which speaks as much for the efficacy of Wildbad as its powers in curing disease does. I mean its injurious influence on the healthy. This influence is perceived after a few baths of short duration, or a single bath of long duration, especially in young persons who are plethoric, and inclined to congestions. In one case the symptoms threatened apoplexy; in others a simple but active vascular fever came on, which was accompanied by oppressive headach, lasted for weeks, and was resolved by perspiration. In some persons the local application of our springs, as a hip or foot-bath, has caused a sensation of warmth in the skin, lasting more than twelve hours, and was like that which follows a sinapism, without producing redness of the skin. The patients asserted that they had not remarked it after local baths of common water at the same temperature, nor even after the use of entire baths of the Wildbad springs. The reason of this may have been, at least in the latter case, that a point of comparison, furnished by the unmoistened part of the skin, was wanting.

The season at Wildbad begins on the 15th of May.—*London Medical Gazette, from Zeitschrift fur die gesammte Medicin*, May, 1840.

